# A fuzzy based dietary clinical decision support system for patients with multiple chronic conditions (MCCs)

**DOI:** 10.1038/s41598-023-39371-4

**Published:** 2023-07-27

**Authors:** Leila Marashi-Hosseini, Sima Jafarirad, Ali Mohammad Hadianfard

**Affiliations:** 1 Department of Health Information Technology, School of Allied Medical Science, Ahvaz Jundishapur University of Medical Sciences, Ahvaz, Iran; 2 Associate Professor of Nutrition and Metabolic Diseases Research Center, Clinical Sciences Research Institute, Ahvaz Jundishapur University of Medical Sciences, Ahvaz, Iran; 3 Associate Professor (Medical Informatics), Nutrition, and Metabolic Diseases Research Center, Clinical Sciences Research Institute, Ahvaz Jundishapur University of Medical Sciences, Ahvaz, Iran

**Keywords:** Type 2 diabetes, Information technology

## Abstract

Due to the multifaceted nature of Multiple Chronic Conditions (MCCs), setting a diet for these patients is complicated and time-consuming. In this study, a clinical decision support system based on fuzzy logic was modeled and evaluated to aid dietitians in adjusting the diet for patients with MCCs. Mamdani fuzzy logic with 1144 rules was applied to design the model for MCCs patients over 18 years who suffer from one or more chronic diseases, including obesity, diabetes, hypertension, hyperlipidemia, and kidney disease. One hundred nutrition records from three nutrition clinics were employed to measure the system's performance. The findings showed that the diet set by nutritionists had no statistically significant difference from the diet recommended by the fuzzy model (p > 0.05), and there was a strong correlation close to one between them. In addition, the results indicated a suitable model performance with an accuracy of about 97%. This system could adjust the diet with high accuracy as well as humans. In addition, it could increase dietitians' confidence, precision, and speed in setting the diet for MCCs patients.

## Introduction

Today, nutrition informatics and intelligent systems aid dietitians by providing some recommendations on setting diets and play an influential role in enhancing diet therapy and clinical practices^1,2^. Intelligent systems based on mathematical approaches, such as fuzzy logic, can be mentioned among these systems. Fuzzy logic models are close to human thinking and reasoning. Therefore, the fuzzy theory is a suitable tool for modeling complex systems where data are insufficient, vague, or unclear^3,4^. Fuzzy logic, in contrast to Aristotelian two-valued logic (yes or no, true or false), is a multi-valued logic where an object can be a member of a set by the degree of membership (from 0 to 100 percent). Since 1965, when Lotfi Alizadeh proposed this approach, the fuzzy model has been applied widely and expanded in nutrition science. Literature indicates that there are many studies in designing intelligent models based on fuzzy inference to recommend the diet for chronic diseases such as diabetes^5,6^, blood pressure^7^, dyslipidemia^8^, kidney diseases^9^, and other conditions^10,11^. In addition, fuzzy logic has been used to design decision support systems (DSS) as powerful tools for helping to make decisions in adjusting the diet for patients with multiple chronic conditions (MCCs)^12^.

MCCs state a patient with two or more chronic diseases simultaneously^13,14^. MCCs usually last more than a year, causing daily activities limitations and requiring continuous medical care^15^. These diseases are not just a set of several distinct conditions. Instead, they represent chronic conditions that often have common underlying causes. When they occur together, they can severely affect the care plan and treatment outcomes as well as the patient's quality of life^16^. Studies have shown that one in every three lives with MCCs globally, which is increasing dramatically^14,17,18^. For example, renal diseases, diabetes, hypertension, hypercholesterolemia, and obesity are recognized as the most common chronic conditions in medical texts^19–23^. Of these, diabetes is a high-prevalence disease in which more than 40% of patients have three or more comorbidities^24^. In addition, it is the most cause of chronic kidney disease, in a way that poorly-controlled hyperglycemia has been associated with the development and progression of chronic kidney diseases. Also, almost 58 to 70% of the patients with diabetes have hypertension, a symptom that causes renal disease. However, these conditions often require a diet to prevent disease progression and guarantee quality of life^25^. For instance, adherence to a diet to control fat is a non-pharmaceutical treatment for hypertension^26^.

Lifestyle changes and inappropriate behavior, such as unhealthy diet and lack of physical activity, can cause appearing chronic conditions^27^. Malnutrition, inefficient management of MCCs, limitations caused by some chronic diseases, and prescribed drug interactions are among the factors leading to increased costs for implementing nutritional and clinical guidelines for patients with MCCs. As a result, these patients significantly (1.5 times) less than single-disease patients have access to sufficient, reasonable, healthy, and appropriate food, which can be called food insecurity^28–31^. Therefore, adhering to a healthy lifestyle, including a healthy diet and physical activity, can increase the life expectancy of patients with more than one chronic disease by 7.6 years in females and 6.3 years in males^32^. A healthy diet is defined as a diet that includes the right and balanced amount of macronutrients (carbohydrates, proteins, and fats) and micronutrients (vitamins and minerals) and provides enough water^33^. Dietary guidelines such as USDA My Plate (United States Department of Agriculture) and Harvard Healthy Eating Plate discern the type and amount of food consumption from five major food groups (bread and cereal, milk and dairy products, meat and proteins, fruits and vegetables) based on the age and physical activity level of the individual^34^. These guidelines follow a protective diet, enabling individuals to make better decisions to prevent malnutrition, obesity, and chronic conditions such as hypertension and diabetes^35^.

It should not be overlooked that a proper diet can effectively help the treatment of some chronic diseases, and it also should be noted that there are differences between setting diets for patients with MCCs and patients with a single disease^36^. For example, for a patient with diabetes and hypercholesterolemia at the same time, it is complicated to set a low-fat and low-carbohydrate diet that can cover both problems^37^. In addition, determining a daily consumption range (maximum and minimum) of micronutrients and macronutrients depends on factors such as age, gender, physical condition and activity, and the presence or absence of diseases. Therefore, it isn't easy to provide a suitable diet using the classical approach^38^.

Obviously, setting a special diet for each patient based on their needs, which relies on accurate calculations of the energy, carbohydrates, protein, and fat requirements, is a complex and time-consuming task^39^. Therefore, a decision support system that can integrate the best clinical evidence with the best practice for severe conditions when patients involve in multiple chronic diseases, as well as improve the quality of care and the decision-making of clinicians, can be a suitable solution^40^. As far as we know, these systems were primarily developed for drug treatments, errors, and drug interactions caused by the prescription of multiple drugs^41–45^, and clinical guidelines and diagnoses^45^. In nutrition and diet, most systems focus on single disease management (for example, obesity, diabetes, and chronic kidney disease)^46–49^. In addition, most diet management apps have been designed for the self-care of patients with MCCs^50^.

To the best of our knowledge, studies have shown the lack of intelligent decision support systems that help dietitians to arrange an appropriate diet according to patients' clinical and anthropometrics data and MCCs in that patients are involved^51^. Therefore, this study aimed to model a knowledge-based clinical decision support system to help dietitians to set a diet that included the optimal number of macronutrients and food groups for patients with MCCs using Mamdani's fuzzy inference system.

## Methods

### Inclusion and exclusion criteria

In this study, a clinical decision support system (CDSS) based on the fuzzy inference system was modeled to help dietitians to set an optimal diet for MCCs patients. The data of adult patients (over 18 years old) who simultaneously suffer from one or more chronic diseases, including obesity, diabetes, high blood pressure, hypercholesterolemia, hypertriglyceridemia, and nephrotic syndrome or chronic kidney disease (before dialysis or hemodialysis or peritoneal dialysis) were applied to model the system. The data of pregnant women, people with disability, patients who recently underwent a surgical procedure, and athletes with specific diets were excluded from this study. Although this was not an interventional or clinical trial study, all methods were carried out under the relevant guidelines and regulations, and informed consent was obtained from all participants (the form in Persian mode is available). Also, the study protocol with the reference number of IR.AJUMS.REC.1400.433 was approved by the Ethics Committee of Ahvaz University of Medical Science.

### Knowledge base system development

In order to enter patients' demographic data, including age, gender, current weight, height, physical activity level, and diseases (conditions), and calculate the indicators presented in Table 1, a web-based platform was developed.

**Table 1:** The formulas to calculate anthropometric indices.

Indicators	Calculation formulas*
Body mass index (BMI)	Weight (kg)/Height _(m)_^2^
Ideal body weight _(kg)_	Ideal BMI × Height _(m)_^2^
Adjusted ideal body weight (ADIBW)	IBW + 0.25 × (Actual weight—IBW)
Basal energy expenditure (BEE) in woman	ADIBW × 1 × 24
Basal energy expenditure (BEE) in man	ADIBW × 0.95 × 24
Energy expended in physical activity (EEPA)	Sedentary (0.30) × BEE
Thermic effect of food (TEF) calories	(BEE + EEPA) × 0.10
Total daily energy expenditure (TEE)	BEE + EEPA + TEF
Daily protein intake	Weight × Recommended amount of protein
Percentage of calories from protein	(TEE ÷ (4 × Daily protein intake)) × 100

*The formulas were sourced from the well-known nutrition science textbook, "Krause and Mahan's Food and the Nutrition Care Process"^35^.

Adjusted total daily energy expenditure was also defined, as shown in Table 2.

**Table 2:** Adjusted total daily energy expenditure formula.

Body mass index (BMI)	Adjusted total daily energy expenditure (TEE)*
Less than 18.5	TEE + 500 _(kcal)_
18.5 to less than 25	TEE
25 to less than 30	TEE – 500 _(kcal)_
30 or greater	TEE – 700 _(kcal)_

*Extracted from reference number^52^.

Both literature and input from experts were employed to develop the knowledge base of the model and make fuzzy rules (if–then statements). In the first step, a review of literature such as articles, textbooks, and guidelines was conducted to determine the appropriate percentages of macronutrients (carbohydrates, proteins, and fats) requirement for each disease. In the second step, a questionnaire was developed based on the results obtained from the literature and then distributed to twelve qualified nutritionists to gather their professional viewpoints. The questionnaire aimed to establish the appropriate percentage of macronutrients in the diet based on the body mass index for each disease. In addition to expert opinions, three nutrition science professors commented on the results obtained from the questionnaire. In the next step, the results of the questionnaire were utilized to develop fuzzy rules and design the model as follows:

- The average of macronutrient percentages expressed in the questionnaire by the experts was calculated and used in the fuzzy rules.
- For patients with kidney diseases, the weight variable was employed as an input in the fuzzy rules.
- In the case of non-kidney diseases, the binary variable of obesity (with values of either obesity or non-obesity) was used instead of weight.

The exact energy percentage (kcal) from proteins in renal disorders was calculated according to Table 1. The recommended protein intake in patients with the renal disease was considered 0.75 g in pre-dialysis, 1.2 g in hemodialysis and peritoneal dialysis, and 1 g in nephrotic syndrome and diabetic nephropathy patients. Also, the linguistic values for other outputs were defined based on the nutrient substitution table of non-renal and renal patients (pre-dialysis, hemodialysis, and peritoneal dialysis)^53^ with the supervision and guidance of a nutritionist.

**Figure 1:**
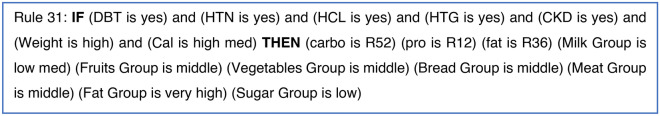
An example of the fuzzy rules.

As shown in Table 3, eleven variables and ten variables applied as the input and output, respectively. The following formula was used to determine the number of fuzzy rules.

$${\text{I}}\, = \,{\text{k1}}\, \times \,{\text{k2}}\, \times \,...\, \times \,{\text{kn,}}$$

where ‘I’ represents the total number of rules, ‘n’ is the count of variables, and ‘k’ is the number of linguistic values for each variable. Therefore, 1144 fuzzy rules were developed to model the system. An example of the rules is shown in the Fig. 1.

**Table 3:** The list of input/ output variables.

Input variables			
Name	Type	Numerical value	Linguistic value
Obesity	Binary	0–1	Yes–No
Diabetes	Binary	0–1	Yes–No
High blood pressure	Binary	0–1	Yes–No
Hypercholesterolemia	Binary	0–1	Yes–No
Hypertriglyceridemia	Binary	0–1	Yes–No
Nephrotic syndrome	Binary	0–1	Yes–No
Chronic kidney disease (pre dialysis)	Binary	0–1	Yes–No
Chronic kidney disease (hemodialysis)	Binary	0–1	Yes–No
Chronic kidney disease (peritoneal dialysis)	Binary	0–1	Yes–No
Weight	Numerical (Range)	(< 40 kg)–(80 kg <)	Very low–Low–Low med–High med–High–Very high
Energy expenditure	Numerical (Range)	(< 1000 kc)–(2600 kc <)	Very low–Low–Low med–High med–High–Very high

Output variables			
Name	Type	Value	Linguistic variable
Carbohydrates	Numerical (Range)	(< %52)–(%58 <)	Less than 52 to Greater than 58
Protein	Numerical (Range)	(< %9)–(%18 <)	Less than 9 to Greater than 18
Fat	Numerical (Range)	(< %24)–(%38 <)	Less than 24 to Greater than 38
Milk and dairy products	Numerical (Servings range)	(< 0.75)–(3.25 <)	Very low–Low–Low med–Middle–High med–High–Very high
Fruits	Numerical (Servings range)	(< 2.5)–(5.5 <)	Very low–Low–Middle–High–Very high
Vegetables	Numerical (Servings range)	(< 2.5)–(5.5 <)	Very low–Low–Middle–High–Very high
Bread and cereals	Numerical (Servings range)	(< 5.5)–(11.5 <)	Very low–Low–Middle–High–Very high
Meats and other protein foods	Numerical (Servings range)	(< 1.5)–(7.5 <)	Very low–Low–Middle–High–Very high
Fat and oils	Numerical (Servings range)	(< 0.5)–(10.5 <)	Very low–Low–Low med–Middle–High med–High–Very high
High calorie foods and sugar	Numerical (Servings range)	(< 0.5)–(5.5 <)	Very low–Low–Middle–High–Very high

Table 3 illustrates the list of input and output variables and their types, numerical values and linguistic values.

### Architecture of the fuzzy model

The fuzzy model was developed in MATLAB R2020b. Since the model's outputs were fuzzy sets, the Mamdani inference method was employed as the inference engine and used for the defuzzification stage. The Mamdani method is a type of fuzzy inference system suitable for outputs that are fuzzy sets, while the Sugeno method is utilized for output membership functions that are either linear or constant. The Mamdani method has proven to be useful in health DSSs due to the visual and interpretative nature of the rules. It is executable in both multi-input and multi-output (MIMO) and multi-input and single-output (MISO) scenarios^54^. In addition, the Gaussian Membership Function was employed to determine the membership values of input and output variables.

To improve the speed and accuracy of the model, it was divided into five sub-models based on the number of simultaneous conditions, including Sub-model no. 1 had 84 rules when there was one condition, sub-model no. 2 had 296 rules when there were two conditions simultaneously, and sub-model no. 3 has 424 rules for three simultaneous conditions, sub-model No. 4 has 276 rules for four simultaneous conditions, and sub-model No. 5 has 64 rules for when there are five simultaneous conditions. This implies that the model was designed to cater to scenarios with a maximum of 5 concurrent conditions. Moreover, we used the centroid approach for fuzzification.

**Figure 2:**
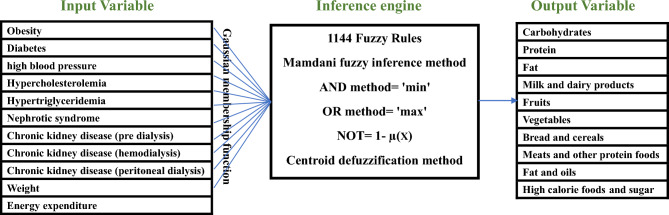
The diagram of the dietary model based on the Mamdani fuzzy inference system.

Figure 2 provides a general view of the model.

### System evaluation

A web-based platform was designed to implement and test the model. Therefore, the information related to 100 nutritional records was entered into this platform. Data were extracted from 100 nutrition records of the different patients with more than one chronic condition (including obesity, diabetes, high blood pressure, hypercholesterolemia, hypertriglyceridemia, and nephrotic syndrome or chronic kidney disease) referred to the three nutrition clinics in Ahvaz (a metropolitan in the southwest of Iran). Also, to measure the system's performance, the average diet set by the three nutritionists was considered the gold standard. Due to the normality of the distribution of the variables not confirmed by Kolmogorov–Smirnov test (p < 0.05), the system's output was compared with the gold standard using the non-parametric Wilcoxon test. Additionally, the correlation between the variables was examined using the Pearson correlation coefficient in SPSS 26, with a significance level of 0.05. Furthermore, the operating characteristics, including sensitivity, specificity, and accuracy, were evaluated to measure the model's performance.

## Results

36% of the participants were Female, and 64% were Male. Table 4 shows the attributes of 100 patients participating in the study. The range of age was from 20 to 80 years, with a mean of 53.4 (± 1.302; α = 0.05).

**Table 4:** Frequency distribution of the participants based on the number of conditions they had, sex, age, and BMI.

Concurrent chronic conditions					Sex		Age			BMI			
1	2	3	4	5	Male	Female	20–40	40–60	60–80	< 18.5	18.5–25	25–30	> 30
19%	48%	22%	9%	2%	36%	64%	13%	51%	36%	4%	27%	35%	34%

Results of the Wilcoxon test, a nonparametric statistical test, show in Table 5. The results indicated no statistically significant difference between the recommended diet by the nutritionists and the diet sat by the model, and they were nearly the same (p > 0.05).

**Table 5:** The agreement between the diet recommended by the nutritionists and the diet adjusted by the fuzzy model.

Nutrients in the diet	Pearson correlation coefficient	P-value* (Pearson)	P-value* (Wilcoxon test)
Adjusted total daily energy expenditure	0.966	0.000	0.414
Carbohydrate	0.998	0.000	0.317
Protein	0.998	0.000	0.564
Fat	0.998	0.000	0.157
Milk and dairy products	0.986	0.000	1.00
Fruits	0.979	0.000	1.00
Vegetables	0.975	0.000	0.564
Bread and cereal	0.993	0.000	0.317
Meats	0.985	0.000	0.157
Fats and oils	0.986	0.000	0.180
Sugar or high-calorie foods	0.988	0.000	0.157

*The significance level was considered 0.05.

Likewise, Table 5 shows the results of the Pearson bivariate correlation test. The results presented that there was a strong correlation (close to one) between the diet recommended by nutritionists and by the fuzzy model (p < 0.05).

In addition, results indicated a suitable performance for the model, as shown in Table 6.

**Table 6:** The characteristics operation of the fuzzy model.

Nutrients in the diet	Accuracy	Sensitivity	Specificity
Adjusted total daily energy expenditure	94	95.9	97.9
Carbohydrate	99	100	99
Protein	97	97.9	98.9
Fat	98	100	98
Milk and dairy products	96	97.9	97.9
Fruits	98	98.9	98.9
Vegetables	97	97.9	98.9
Bread and cereal	99	99	100
Meats	98	98	100
Fats and oils	95	95.9	98.9
Sugar or high-calorie foods	98	100	98

## Discussion

This study presented a high-accuracy DSS to assist nutritionists in sitting diet for patients with MCCs. The system was able to estimate the number of macronutrients and main food groups required by each patient. Also, it was able to determine the healthy range of each food group based on nutritional guidelines and the patient's chronic conditions. In particular, for patients with diabetes or chronic kidney disease, a balanced diet intake of carbohydrates, protein, and fat is essential to control and treat their conditions.

Studies showed that the amount of energy required from proteins in patients in the pre-dialysis stages was calculated according to their weight and is about 0.6 to 0.8 of the patient's ideal weight^55–57^. In addition, another study recommended that the number of macronutrients, including carbohydrates, protein, and fat, should be considered as 49–54%, 19–20%, and 21–26%, respectively^58^. Moreover, the results showed that the number of macronutrients provided by the model was consistent with these studies. Also, the model's outputs followed nutritional guidelines such as USDA My Plate and Harvard Healthy Eating Plate^59^.

Since total energy expenditure forms the foundation of an optimal diet, the system avoids the arbitrary determination of this parameter and recommends its optimal value to the dietitians by accurately calculating the patients' total daily energy expenditure via BMI.

Fuzzy logic is one of the most appropriate ways to solve the problem of Multiple-Criteria decisions^60^. Therefore, the fuzzy approach has been used in many studies about dietary recommendations. A well-known example of the application of the fuzzy model was called 'Leemoo'. it could plan and assess various diets by providing a broad range of recommended meals. In a similar way, the model proposed in this study, it also could compute the amounts of nutrients and food group intakes and compare them with recommendations of Dietary Reference Intakes and MyPyramid guideline. In addition, it recommended a balanced consumption range instead of an optimal consumption point^61^. However, in contrast with the present model, it has been designed for healthy people. Another study introduced a diet recommendation system based on artificial neural networks and Sugeno fuzzy logic methods. Unlike the present model, it had only one output and selected the appropriate diet from only 11 meal plans^62^.

In addition to the fuzzy model, other algorithms have been used to set diets for patients with chronic diseases. For instance, a decision tree hierarchical model was applied to design a dietary recommendation system for chronic conditions patients called DIETOS. Similar to the current model, it was proposed to aid nutritionists with 100% specificity and 91% sensitivity^63^. Also, a DSS named Jena was designed based on ontology and decision tree algorithms to help nutritionists recommend the diet for patients with chronic renal diseases, and an accuracy of 100% was reported for it^64^. Although these studies showed higher accuracy with the decision tree method than the present study, they were designed only for patients with chronic kidney diseases and could not use for MCCs.

Furthermore, some studies have concentrated on self-care in patients with MCCs. For example, 'SousChef' was a mobile-based food recommendation system elderly with MCCs. It employed heuristic functions to recommend a personalized diet based on information provided by the patient, including anthropometric data, personal preferences, and physical activity level. Also, a survey showed 70% satisfaction with the system^65^. Nevertheless, this system was not designed to help dietitians.

However, the novelty of this study was to propose a model to help dietitians adjust the diet for patients with MCCs by calculating the number of macronutrients according to daily energy consumption, weight, the conditions that the patient is suffering from, dietary guidelines, and nutrient substitution table.

To the best of our knowledge, no similar study has been found, and most studies in dietary recommendations focused on patients with a single disease or healthy individuals rather than MCCs. Therefore, finding similar studies that support the results was one of the limitations of this study.

Another challenge in designing and measuring the model's performance was determining the gold standard. It was challenging to get a common agreement among nutritionists regarding the exact amount of carbohydrates, protein, and fat. Therefore, the average dietitian's recommendations were used as the gold standard.

Lockdown conditions due to COVID-19 caused obstacles in access to MCCs patients. For this reason, the nutritional records of the patients were employed to evaluate the model performance. Therefore, the incomplete nutritional patient records, as well as the variety in diet recommendations, were other study limitations. As a result, two other dietitians adjusted the diet to guarantee the accuracy of each diet.

## Conclusion

The results suggested the fuzzy logic algorithm can be a suitable method in designing CDSSs for diet recommendation. The proposed system enhanced the reliability, speed, and accuracy of the decision-making of dietitians in setting an optimal diet for patients with MCCs. The system can further be extended to include the rules related to the intake of micronutrients and fluids. In addition, by adding other MCCs and developing its knowledge base, this system can be helpful in clinical decision-making and providing suitable diets for different patients.

## Data Availability

The datasets used and/or analyzed during the current study are available from the corresponding author upon reasonable request.
